# Tick-borne viruses and their risk to public health in the Caribbean: Spotlight on bats as reservoirs in Cuba

**DOI:** 10.1016/j.heliyon.2024.e26118

**Published:** 2024-02-09

**Authors:** Maritza Pupo Antúnez, José Carlos Marín Montesinos, Alexandra Corduneanu, Dasiel Obregón, Sara Moutailler, Alejandro Cabezas-Cruz

**Affiliations:** a Laboratorio de Virología. Departamento de Microbiología y Virología. Facultad de Biología, Universidad de la Habana, C.P. 10400, Plaza de la Revolución, Cuba; b Department of Animal Breeding and Animal Production, University of Agricultural Sciences and Veterinary Medicine of Cluj-Napoca, Cluj-Napoca, Romania; c Department of Parasitology and Parasitic Diseases, University of Agricultural Sciences and Veterinary Medicine, Cluj-Napoca-Napoca, Romania; d School of Environmental Sciences, University of Guelph, Guelph, ON N1G 2W1, Canada; e Anses, INRAE, Ecole Nationale Vétérinaire d’Alfort, UMR BIPAR, Laboratoire de Santé Animale, Maisons-Alfort, F-94700, France

**Keywords:** Ticks-borne virus, Ectoparasites, Reservoirs, Bats

## Abstract

In recent decades, tick-borne diseases (TBDs) have surged and expanded globally due to factors like changes in human activities, land use patterns, and climate change, and it have been associated with the emergence of zoonotic diseases. Cuba faces the impact of ticks on human health and the economy. Although Cuba has studied TBDs extensively for the past 50 years, focus on tick-borne viral pathogens affecting humans remains scant. Despite TBDs not currently being a major health concern in Cuba, factors like inadequate clinician awareness, climate conditions, global tick emergence, and evidence of zoonotic pathogens in ticks underscore the importance of enhanced TBD surveillance in the country. Here we revised the available information on ticks as vectors of pathogenic viruses to humans, spotlighting bats as potential reservoirs of tick-borne viruses (TBVs). Ticks on bats have gained interest as potential reservoirs of pathogenic viruses to humans in Cuba and worldwide. Understanding their role in maintaining viruses and their potential transmission to humans is crucial for the implementation of surveillance and control programs to reduce the risk of tick-borne viral diseases and public health management.

## Highlights

• Bats play a crucial role as hosts for diverse viruses, some of which are transmitted by ticks.
• Rich bat diversity in the Caribbean, presents opportunities for virus maintenance and cross-species transmission.
• Examination of bat-associated ectoparasites, especially ticks, reveals their potential as reservoirs for viral pathogens.
• Tick-borne viruses, belonging to multiple families, have the potential for epidemics and zoonotic transmission.
• Understanding the dynamics of bat-borne viruses in ticks is essential for mitigating public health risks.

## 1 Introduction

Ticks are obligatory hematophagous arthropods of great economic and health importance, as they affect the health of humans and animals worldwide. They are divided into three families: Nuttalliellidae (with a single genus and species), Ixodidae or hard ticks (with 14 genera and approximately 700 species), and Argasidae or soft ticks (including 5 genera and 200 species). Around 10% of the known species are capable of transmitting pathogens such as viruses, bacteria, and protozoa [1], making them excellent vectors for arboviruses, second only to mosquitoes [2].

In recent years, tick-borne diseases (TBDs) incidence and prevalence have increased and spread to new areas around the world. This phenomenon has been attributed to changes in human activities, demographic shifts, land use [3], rising temperatures, variations in precipitation, and extreme weather events as potential consequences of climate change [4], resulting in the emergence and re-emergence of zoonotic diseases [3].

In Cuba, 34 species of ticks have been described, with four from the Ixodidae family—*Rhipicephalus microplus* (primarily affecting cattle), *Rhipicephalus sanguineus* (affecting dogs), *Dermacentor nitens* (associated with horses) and *Amblyomma mixtum* (having a broad spectrum of mammalian hosts)—considered particularly significant in terms of both human health and the economy [5]. Over the past 50 years, various institutions have been actively engaged in eco-epidemiological studies, prevention, and control of TBDs [5]. Despite this, the majority of research pertaining to human health have focused on bacteria and protozoa, with limited attention given to understanding the potential role of these vectors in transmitting pathogenic viruses to humans.

Although TBDs are not currently considered a health problem in Cuba, there are factors that justify and make the surveillance of TBDs necessary. These factors include limited information and recognition among clinicians about tick-borne zoonoses, the country's climate conditions, the global emergence/re-emergence of these vectors, and microbiological, molecular, and serological evidence of zoonotic pathogens in these vectors. Additionally, Cuba is part of global initiatives such as One Health and PREZODE (preventing zoonotic disease emergence), aimed at achieving optimal health for people, animals, and the environment and reducing the risk of zoonotic infectious diseases [3,6,7].

In this context, bats, given their ecological significance, are emerging as subjects of interest in the realm of TBDs. While they play critical roles in various ecosystems, they can also be reservoirs for pathogens, especially when in association with ectoparasites like ticks [[8], [9], [10]].

During the period of Cuban and Czechoslovak cooperation, primary studies on tick diversity in Cuba revealed the highest number of tick species found on free-living vertebrates, with bats, particularly those inhabiting caves, contributing significantly. Notably, soft ticks such as *Antricola marginatus*, *Ornithodoros viguerasi*, and *Ornithodoros kelleyi* were prevalent in these environments. Additionally, *Ornithodoros dusbabeki* was discovered in a palm tree used by bats, specifically *Artibeus jamaicensis*, for night-time roosting [11,12]. A recent article [5] described ticks from the Argasidae family, including species of *Antricola* and *Ornithodoros* found in bat guano. Earlier investigations documented the isolation of the Hughes virus from *Ornithodoros denmarki* ticks parasitizing birds and the Estero real virus from *Ornithodoros tadaridae* ticks parasitizing bats [13]. However, subsequent to these findings, there have been no reported instances of virus isolations from ticks infecting bats.

**Fig. 1:**
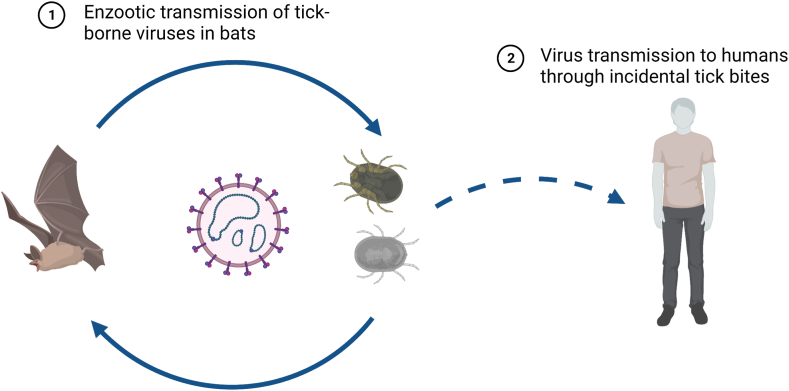
**Interplay between bats and ticks for tick-borne virus transition to humans.** The figure illustrates the dynamic interplay between bats and ticks (1) contributing to the transmission of tick-borne viruses (TBVs) from bats to humans (2), highlighting the key elements in this complex process. Created with BioRender (www.biorender.com).

Given the ecological niches bats occupy and their proximity to human habitats at times, understanding their role as reservoirs, and the tick-borne viruses (TBVs) they might carry, is of paramount importance. The intricate interplay between bat and tick ecology, along with the potential transmission routes of pathogens to humans, forms a complex area of study (Fig. 1). This paper aims to illuminate this intricate subject.

## 2 Methodology

The authors conducted a comprehensive literature search to identify relevant research papers from databases such as PubMed, Scopus, Google Scholar, and Science Direct. The search utilized key terms such as “ticks-borne virus,” “ectoparasites,” “potential reservoirs,” and “bats.” The primary focus for selection criteria was on English language papers, but it also encompassed literature published in Spanish, as well as the earliest available reports and publications accessible in the public domain. A total of 255 papers were initially obtained, and after careful review, 176 were excluded due to insufficient information.

## 3 Ticks-borne diseases: transmission of viruses from ticks to humans

TBDs pose a significant threat to both animal and human health, giving rise to illnesses caused by bacteria, viruses, protozoa, and helminths. This issue has become a global concern [14]. The impact of climate change has facilitated the migration of ticks to new regions, escalating the incidence of TBDs and, in some instances, exacerbating their pathogenesis, posing a growing challenge to both human and animal well-being [15].

Within the tick families, the Ixodidae family stands out as the primary carrier of zoonotic diseases. In Europe, Ixodid ticks play a crucial role as vectors for zoonotic pathogens, affecting a diverse range of livestock. Noteworthy genera include *Ixodes*, *Dermacentor*, *Haemaphysalis*, *Rhipicephalus*, and *Hyalomma*, with *Ixodes ricinus* being the most widespread species [16]. These ticks transmit protozoans such as *Babesia* and *Theileria*, as well as bacteria like *Anaplasma*, *Borrelia*, *Ehrlichia*, and *Neoehrlichia*. Additionally, tick-borne encephalitis virus (TBEV), transmitted primarily by *I. ricinus* and *Ixodes persulcatus* species, is a significant concern [16].

A recent review by de la Fuente et al. [17] highlights the increasing incidence of ticks and TBDs globally, emphasizing the need for understanding the prevalence differences influenced by geographic and climatic variables. While the majority of studies in the Western Hemisphere originate from the USA, followed by Mexico, Central America, and Brazil [17], there remains a lack of epidemiological data in the Caribbean region, despite its high risk of tick and TBDs introduction and dispersal [1].

A total of 56 species of ticks, belonging to 10 genera and 2 families (Argasidae and Ixodidae) including 15 species of *Ornithodoros*, 10 species of *Antricola*, 17 species of *Amblyomma*, 3 species of *Argas*, *Ixodes,* and *Rhipicephalus*, 2 species of *Haemaphysalis*, and 1 species each of *Parantricola*, *Dermacentor* (*Anocentor*), and *Aponomma* have been recorded [18]. The most studied tick species in this region due to the impact on health are those associated with the transmission of tick-borne diseases to livestock or pets. Among them, *R. microplus* is considered as the most important to cattle and is involved in the transmission of bacteria such as *Anaplasma marginale*, *Anaplasma centrale*, and protozoa such as *Babesia bigemina*, *Babesia bovis*, and *Theileria* spp. Other tick species such as *Amblyomma variegatum*, *Amblyomma cajennense*, *D. nitens*, *R. sanguineus* are also prevalent in several Caribbean countries and play an important role in both animal and public health [18].

Cuba, specifically, faces challenges with ticks causing TBDs that affect livestock, and to a lesser extent, human health. Thirty-four tick species have been identified, most within the family Argasidae, but being *R. microplus*, *R. sanguineus*, *D. nitens*, and *A. mixtum* considered the most important in terms of health and economic impact. *R. microplus*, in particular, represents over 80% of ticks infesting cattle and serves as vector for pathogens such as *Babesia bovis*, *Babesia bigemina* and *Anaplasma marginale* to bovines and buffaloes [5].

While Cuba has reported no significant incidence of TBDs in humans, a study from 1998 to 2010 revealed autochthonous infections of *Borrelia burgdorferi sensu lato* (*s.l.*) in individuals suspected of Lyme disease, exposed to tick bites in a province with high tick infestation, mainly attributed to *Amblyomma cajennense*. However, genetic material of *B. burgdorferi s.l.* was not detected in hard ticks with medical and veterinarian importance, though they were not excluded as potential vectors [7,19].

The recognition of ticks as carriers and vectors of pathogenic viruses dates back over a century, marked by the identification of the flavivirus Louping ill virus as the cause of severe encephalitis in sheep [20]. Since then, the prevalence of tick-borne viral diseases (TBVDs) has increased, with the most frequently reported cases often linked to widespread epidemics [20]. The spectrum of identified TBVs encompasses members from 2 orders, 9 families, and 12 genera, alongside other unclassified members [20]. About 25% of these are associated with diseases, and notably, all are zoonotic [21]. Recently, Shah et al. [22] provided a comprehensive review on the subject of TBVs and TBVDs, covering crucial aspects like their epidemiology, pathogenesis, and clinical manifestations. Building on this groundwork, this review will distill the essential aspects of the most significant TBVs and TBVDs in relation to bats, emphasizing their incidence and the associated risks to human populations in the Americas and the Caribbean region.

Most TBVs consist of a diverse group of viruses that circulate between ticks and vertebrate hosts. Taxonomically, most of these viruses have RNA genomes and belong to eight families: Flaviviridae, Orthomyxoviridae, Reoviridae, Rhabdoviridae, Nyamiviridae (order Mononegavirales), and the families Nairoviridae, Phenuiviridae, and Peribunyaviridae (order Bunyavirales). The exception is the African swine fever virus, which belongs to the Asfarviridae family and has a DNA genome. The order Bunyavirales includes nine families of viruses with morphological and genomic similarities, mainly transmitted by arthropods, and many are associated with viral diseases in vertebrates. The main TBVs involve three families from this order: Nairoviridae, Peribunyaviridae, and Phenuiviridae [20].

The Nairoviridae family has a single genus, *Orthonairovirus*, consisting of 35 viruses assigned to 7 approved serogroups as species. These nairoviruses are transmitted by ticks and are associated with natural hosts such as birds, bats, rodents, and other animals. Among them, the Crimean-Congo haemorrhagic fever virus (CCHF) is particularly notorious for its ability to induce haemorrhagic fever in humans. Multiple tick species can serve as carriers for CCHF, with the Ixodes genus being the most efficient, and within it, *Hyalomma marginatum* acting as the primary vector. Other tick species, including *Hyalomma aegyptium*, *Hyalomma schulzei*, *Hyalomma onatoli*, *Hyalomma dromedarii*, *Hyalomma rufipes*, *Hyalomma excavatum*, *Hyalomma anatolicum*, *R. sanguineus*, *Rhipicephalus turanicus*, *Rhipicephalus annulatus*, *Haemaphysalis punctata*, *A. variegatum*, and *Hyalomma truncatum*, among others, also have the potential to transmit the virus [23].

Some of the nairovirus, transmitted by ticks have been found in the Americas. They include virus belongs to Hughes complex. Farallon virus (isolated from *Ornithodorus* spp. from California), [20], Hughes virus, in *Ornithodoros capensis* complex (*O. denmarki* and *O. capensis*) from Trinidad and *O. denmarki* in Cuba, Raza virus from *Ornithodoros* spp. In Mexico, Soldado virus in *O. capensis* complex in Trinidad, and Punta Salinas virus from *O. amblus* ticks in Peru. The Sierra Nevada virus (order Mononegavirales, family Nyamiviridae, genus *Nyavirus*) was isolated from soft ticks *Ornithodoros coriaceus* during a bovine abortion epizootic in California, US, in 1975 [[1], [2], [3]]. It is currently unknown whether it infects mammals and birds naturally.

The Phenuiviridae family, genus *Phlebovirus*, includes the Heartland virus and Lone Star viruses isolated from the lone star tick *Amblyomma americanum* in US [24]. The Heartland virus is endemic in eastern United States and is closely related to the severe fever with thrombocytopenia syndrome virus (SFTSV) and causes fever, thrombocytopenia, leukopenia, fatigue, anorexia, headache, nausea, myalgia, and arthralgia [25,26]. In Latin America, laboratory diagnosis of these viruses is still elementary and extremely difficult. The Peribunyaviridae family, genus *Orthobunyavirus*, is geographically located in Europe, Asia, and Africa.

The Rhabdoviridae family comprises a wide variety of viruses that can affect vertebrates, invertebrates, and plants [27]. Within this family, there are viruses geographically located in the Americas. In addition, transmitted by *A. americanum,* the Long Island tick virus have uncertainties about their pathogenicity in mammals [20,28]. There is also the Sawgrass virus group, including the Connecticut tick, New Minto, and Sawgrass viruses, transmitted by *Ixodes dentatus, Haemaphysalis leporispalustris, Dermacentor variabilis*, *H. leporispalustris* respectively and geographically located in USA which require further studies to clarify their transmission by ticks and their pathogenicity in humans and animals [20].

The Colorado tick fever virus (family Reoviridae, genus *Coltivirus*) was isolated in 1946 from human serum. It has a wide range of hosts and vectors as *Dermacentor andersoni, Dermacentor occidentalis*, *Dermacentor albipictus*, *Dermacentor arumapertus*, *H. leporispalustris*, *Otobius lagophilus*, *Ixodes sculptus*, and *Ixodes spinipalpis*. This virus can cause an abrupt infection in humans with signs such as fever, chills, headaches, and more severe cases leading to central nervous system infection, haemorrhagic fever, pericarditis, myocarditis, and orchitis, mainly in children [20]. The *Orbivirus* genus, within the family Reoviridae (subfamily Sedoreovirinae), includes 5 viruses associated with different bat species located across space and time and phylogenetically associated with different arthropod vectors, such as ticks, indicating a strong association between orbiviruses and bats [8]. Within the *Orbivirus* genus, there are viruses of the Chenuda species, including seven different serotypes found in the genera *Argas* and *Ornithodoros* that parasitize birds. These include Huacho virus, Mono Lake virus, and Sixgun City virus. Although they can cause mortality in birds, there is no evidence of their impact on humans [29,30]. The Chobar Gorge virus, also within the *Orbivirus* genus, is associated with bats and includes two serotypes. It was isolated from *Ornithodoros* spp. ticks in Nepal in 1970. They can infect cattle, horses, and humans [20]. The Great Island virus group comprises members of 36 serotypes, which are widely distributed in Europe, North America, and Eastern Russia. These viruses have been identified in various geographic areas and are associated with ticks of the *I. ricinus* species as the primary vector in birds [31]. The Wad Medani virus, also within the *Orbivirus* genus, includes two serotypes: Seletar and Wad Medani. The latter was isolated from a group of tick larvae of *Amblyomma cajennense s.l.* in Jamaica in 1965 [13]. Currently, it is unknown whether the Wad Medani virus can be pathogenic to livestock and humans [32].

Flaviviruses, belonging to the Flaviviridae family, are transmitted by hematophagous mosquitoes or ticks and have the potential to cause epidemics with significant morbidity and mortality, making them a global health problem. Flaviviruses of medical importance transmitted by mosquitoes include West Nile virus (WNV), Japanese encephalitis virus [33], Yellow fever virus (YFV), Dengue virus (DENV), and Zika virus (ZIKV) [34]. While tick-transmitted flaviviruses have historically garnered less attention due to their comparatively lower incidence in human infections and the greater medical impact of mosquito-transmitted counterparts, the emergence of the Severe Fever with Thrombocytopenia Syndrome virus (SFTSV) in China in 2011 has shifted focus. The escalating incidence and rapid global spread of SFTSV underscore the necessity to explore TBVs. Novel TBVs have been identified using metaviromic approaches [35]. These viruses can infect mammals and birds, and most of the diseases they cause are classified at biosafety levels 3 and 4 [20]. The most representative TBVs are CCHF, TBEV, Powassan virus, Omsk haemorrhagic fever virus, and Kyasanur forest disease virus. The TBEV is the most medically important one. TBE is endemic from Central Europe and the Scandinavian Peninsula to Japan, but the highest incidence has been documented in the Baltic and Central European countries. In the last two decades, an increase in TBE incidence has been observed in endemic areas and the occurrence of sporadic cases outside endemic areas. The incidence of TBE increased to 0.9 cases per 100,000 people in 2020, an increase from rates of 0.7 in 2019 probably as the result of social and environmental factors, increased medical awareness and advanced diagnostics combinations [36].

Most TBVs are reported in Europe, Asia, Africa, and Australia, with the exception of Powassan fever, which is present in the Far East of Russia and North America. In the last one region, the principal vector of prototype POWV is Ixodes cookie tick, especially in the northeastern United States and eastern Canada [37]. In recent years, there has been an increase in human cases reported in North America, attributed to changes in tick transmission [38]. In Mexico, there is a wide diversity of ticks, but the focus has been on detecting bacterial diseases, mainly borreliosis, rickettsiosis, ehrlichiosis, babesiosis, and anaplasmosis. There are few studies on viral agents [39].

Currently, information on TBVs in Latin America is scarce [40]. Most of the knowledge comes from virome studies in ticks using high-throughput sequencing. Advancements in this technology and the growing interest in understanding virome diversity in arthropods have led to the discovery of new viruses in these vectors [1,3]. It is worth to mention that global tick virome is dominated by RNA genome viruses, including single-stranded, double-stranded, positive-sense, or negative-sense, monopartite, or segmented viruses, indicating that ticks provide a unique niche for RNA viruses [35].

In a virome study conducted by Sameroff et al. [41] in Trinidad and Tobago, sequences from 638 ticks were analyzed. The study identified nine viruses, including a novel flavivirus species named Trinbago virus (VTBO), found in three tick species (*R. microplus*, *R. sanguineus s.l.*, and *Amblyomma ovale*). VTBO shares an 86% amino acid similarity with the tick-borne Bole 4 virus identified in China [3], and it has been reported in various locations such as Thailand, Trinidad and Tobago, Slovakia, Romania, and Kenya. Recent phylogenetic analyses have revealed a close genetic association between VTBO and the Haseki virus, previously detected in *Ixodes* ticks in parts of Asia and Russia [41].

Another group of TBVs, the Jingmenviruses, is closely related to flaviviruses but constitutes a distinct genus within the Flaviviridae family. The prototype virus, Jingmen tick virus (JMTV), was discovered in *R. microplus* ticks in China in 2010. Jingmenviruses are separated into two phylogenetic clades: one associated with ticks and vertebrates and the other with insects. JMTV has been detected in various countries and regions across Asia, Europe, Africa, and the Americas, involving a wide range of arthropods as well as mammals, including humans [42]. In humans, JMTV has been linked to sporadic cases of febrile illness in multiple patients [43]. Presently, it is recognized as an emerging arbovirus with a global threat [42].

In conclusion, ticks can transmit a variety of pathogenic viruses to humans, and there is an increasing interest in understanding and identifying these TBVs to mitigate their impact on public health. The tick virome is a rich source of viral diversity, and ongoing research is essential to detect and characterize new TBVs in different regions.

## 4 Bats diversity and their role in pathogen transmission

Mammal diversity in the New World is high [44], especially in the Caribbean islands, which represent an area of high species richness and endemism [45], with around 800 endemic vertebrates [46]. Bats (order *Chiroptera*) make up nearly 20% of all mammal species worldwide [47] and species found in the New World represent one third, with high phylogenetic diversity (e.g., 350 species) [45]. Although, the number of mammal species on an island is smaller than on the mainland, their diversity has been shaped by isolation from their ancestors on the continent [45]. The largest island in the Caribbean is Cuba, with a rich biodiversity and many karst caves (about 3000) [48], which provides roosts for 16 bat species [49]. Currently, Cuba is home to 26 species from 6 families, with eight species being endemic to the island (*Natalus primus*, *Chilonatalus macer*, *Nycticeius cubanus*, *Lasiurus pfeifferi*, *Dasypterus insularis*, *Antrozous koopmanni*, *Mormopterus minutus*, *Phylonypteris poeyi*) [50]. Additionally, eight species have become extinct [49], making it the most bat-diverse region in the West Indies [50]. Bats can colonise all types of habitats, and although the species number of bats in Cuba is not as high compared to Colombian bats (e.g. 209 species) [51], they have a wide dietary diversity (e.g. insectivorous, frugivorous, piscivorous, nectivorous) [50].

Bats are a reservoir for a variety of pathogens, including bacteria [52], parasites [53] and viruses [54]. These mammals can coexist with viruses without clinical signs, and are a source of great viral diversity, including zoonotic viruses with worldwide distribution [54]. Due to their particularities such as a high metabolic rate and body temperature, the ability to fly, particular response of the immune system and torpor, they can maintain and transmit pathogens over a long period of time [55]. The most important viral zoonosis, transmitted from animals of the Order Chiroptera and Carnivora to other animals, including humans, is represented by rabies [56]. This disease attacks the nervous system of the host and is responsible for more than 55,000 deaths worldwide and is a major health, economic and environmental problem [57]. Rabies is caused by a negative-stranded RNA virus from the genus *Lyssavirus* (family Rhabdoviridae) [58]. The virus is distributed worldwide, and comprises 13 recognized species: *Rabies virus* (RABV), which affects a wide range of species, including bats in the New World and carnivores around the globe. Among them, *Lagos bat virus* (LBV), detected in frugivorous (pteropodid) bats, cats and dogs [59]; *Mokola virus* (MOKV) in shrews, rats, cats, dogs and humans [60]; *Duvenhage virus* (DUVV) in insectivorous bats with human cases reported [61]; *European bat-lyssavirus*-1 (EBLV-1), in insectivorous bats (*Eptesicus serotinus* and *E. isabellinus*) [62]; *European bat-lyssavirus*-2 (EBLV-2), in insectivorous bats from *Myotis* genus and human cases reported [63]; *Australian bat lyssavirus* (ABLV), in frugivorous and insectivorous bats, humans [64]; *Irkut virus* (IRKV): insectivorous bats (*Murina leucogaster*) and in humans [65]; *Aravan virus* (ARAV), *Khujand virus* (KHUV): insectivorous bats from *Myotis* genus from Central Asia; *West Caucasian bat virus* (WCBV) from *Miniopterus schreibersii* in South-Eastern Europe [66]; *Shimoni bat lyssavirus* (SHIBV) reported from *Hipposideros commersoni* [67] and *Bokeloh bat lyssavirus* (BBLV) from *My. natterii* in Germany [68].

While in Europe, Africa, Asia and Australia bats (especially insectivorous and frugivorous) are reservoirs for a variety of lyssavirus species, in the New World hematophagous and insectivorous bats are reservoirs only for the widespread RABV [67]. In Latin America and on most Caribbean islands, rabies is mainly transmitted by the vampire bats [69]. Despite the fact that there are 3 species of vampire bats, *Desmodus rotundus* is more commonly involved in outbreaks in both animals and humans [69,70]. In the Caribbean region, rabies is a notifiable disease on the islands of the Dominican Republic, Haiti and Cuba and active surveillance of the disease is carried out [71]. The wildlife reservoir of RABV in the islands is mainly represented by the Indian mongoose (*Herpestes auropunctatus*) (Cuba, Grenada and Puerto Rico) [71,72], while vampire bats represent a reservoir in the south of the Caribbean islands and on the mainland of Latin America (Trinidad and Tobago, Guyana, Suriname, Belize and French Guiana). In Cuba, the first case of rabies was reported in mongoose in 1956 and later in carnivores and insectivorous bat species such as *Eptesicus fuscus* and *Eumops glaucinus* [71,72]. The Cuban rabies isolates of terrestrial species form a single monophyletic group [73]. There are limited data on the presence and characterisation of lyssavirus in the Caribbean islands and further strategies need to be implemented to prevent bat-transmitted cases in humans.

## 5 Ticks on bats, potential reservoirs of pathogenic viruses to humans

Research on bat-transmitted viruses has gained interest in recent years due to the occurrence of epizootics and epidemics caused by viruses that use these mammals as hosts. Bats are exceptional in their ability to act as natural reservoirs of viruses, and they are able to harbour more diverse viruses per animal species than any other mammalian order [55]. Several high impact zoonotic disease outbreaks in the last two decades have been linked to bat-borne viruses, including Severe Acute Respiratory Syndrome Coronavirus (SARS-CoV) and Middle East Respiratory Syndrome Coronavirus (MERS-CoV) [74].

Bats are the only mammals that fly, which assists in virus dispersal, and they roost in large numbers, which increases the likelihood of interspecies transmission. In addition, the bats extreme longevity enhances the potential for bats to maintain persistent infection and transmit viruses [54]. In addition to serving as viral reservoirs, bats also host a variety of ectoparasites, including ticks. These arthropod ectoparasites can act as vectors for transmitting pathogens, such as viruses, to humans and other mammal species [75]. Although bats may be resistant to infections with ectoparasite-borne viruses, these viruses can still be infectious or pathogenic to other mammals, including humans, and can be transmitted through incidental bites from bat ectoparasites [9].

Despite the recognized importance of bats in ecosystem dynamics and disease transmission, the role of their ectoparasites in harbouring and transmitting viruses within bat populations, and potentially to humans or other mammals, remains underexplored. Given the high specialization and diversity of bat ectoparasites, there is a strong possibility that they function as reservoirs for specific viruses, effectively sustaining these pathogens within bat communities [76]. Transmission pathways are multifaceted: bats, for instance, may ingest ectoparasites during grooming, either their own or those from conspecifics, thereby facilitating the spread of pathogens. The symbiotic relationship bats share with arthropods, particularly as many bat species are insectivores, further exposes them to a diverse array of insect-borne viruses [77]. Beyond ingestion, vector-borne transmission, particularly through bites, stands as a dominant mechanism for pathogen spread [76]. Among the blood-sucking arthropods that infect bats are bat flies and wingless bat flies, as well as non-obligate parasites such as ticks, mites, and fleas [78]. Ticks naturally host a wide variety of viruses, bacteria, and protozoa. Since ticks are obligate blood feeders, these parasitic arthropods can also carry viruses acquired from their hosts during blood meals [79]. Ticks parasitizing bats are of particular interest as bats are potential natural reservoirs of pathogens relevant to public and veterinary health.

For example, a study on the soft tick *Carios vespertilionis* found a remarkable diversity of RNA viruses and bacteria present in these ticks, highlighting the importance of bat-associated ectoparasite surveillance as an effective and non-invasive means to track viruses and bacteria circulating in bats and ticks [80]. Another study investigated the viral composition of adult *Antricola delacruzi* ticks collected in a bat cave in the Western Amazonia of Brazil, contributing to the understanding of the viral diversity in bat-associated ticks [81].

Furthermore, a study on Kasokero virus (KASV) suggests that this virus is maintained in an enzootic transmission cycle involving *Ornithodoros (R.) faini* ticks and *Rousettus aegyptiacus* bats, indicating that these ticks have the potential to be vectors for virus spillover into humans [82]. Additionally, a study on bat-specialist ticks in eastern Europe found that *Carios vespertilionis* and *Secretargas transgariepinus* are known to be competent vectors for a series of viral, bacterial, and protozoan pathogens, some of which are relevant to public and veterinary health [80].

Lastly, research on Crimean-Congo haemorrhagic fever-like viruses in African bats found evidence for widespread infection, with bats commonly infested with soft and hard ticks, and the specific habitat conditions in caves potentially enabling a virus amplification cycle between bats and ticks [83]. These examples provide evidence of viruses being transmitted by ticks from bats, emphasizing the need for continued research and surveillance of bat-associated ectoparasites. Further research in this area will contribute to a better understanding of the dynamics of bat-borne viruses and aid in the development of strategies to mitigate the risk of spill over events.

## 6 Discussion

Ticks are significant vectors of pathogenic viruses to humans and animals, contributing substantially to the global burden of TBDs. The increasing incidence and widespread geographical distribution of TBDs underscore the need for comprehensive surveillance and control measures. Approximately 10% of known tick species can transmit pathogens, positioning ticks as major contributors to arbovirus transmission, second only to mosquitoes.

The diversity of viruses within tick populations, including various families of RNA viruses, highlights the complex interactions between ticks, vertebrate hosts, and the pathogens they carry. Furthermore, bats as potential reservoirs of pathogenic viruses transmitted by ticks, further adds complexity to tick-borne infections. Bat interactions with ectoparasites, such as ticks, can facilitate pathogen transmission.

Our review highlights the importance of ticks as vectors of pathogenic viruses to humans, and particularly ticks parasitizing bats, indicating a risk for the health of animal populations, including humans. Ticks on bats have gained interest as potential reservoirs of pathogenic viruses to humans in worldwide.

Although research in TBVs from bats in the Latin America is scarce, significant progress has been made in recent years due to the use of molecular techniques [84]. Noteworthy regional events, such as the Pre-REDIPRA Seminar “Rabies: pending challenges – an unfinished process”. Guatemala, 2017 (PAHO) [85] and the II Congress of the Latin American Society for Vector Ecology “Control of endemic zoonotic and vector-borne emerging and re-emerging diseases, Argentina 2022 [86], have focused on bats as important reservoirs for infectious diseases. The former emphasized the investigation of RABV in non-vampire bats in the Caribbean due to the rising incidence of bat-transmitted rabies in Latin America, which currently exceeds cases of canine-transmitted rabies. The latter highlighted the serological evidence of hantavirus in bats and the record of eleven human rabies deaths involving hematophagous bats in Brazil. Health networks have also been established in the Caribbean to mitigate the emergence of animal and zoonotic infectious diseases and their significant economic impact [1].

Although TBDs and TBVs are not a major health concern in Cuba, the presence of zoonotic pathogens in ticks and changing environmental conditions necessitate ongoing monitoring and research. Particularly noteworthy is the fact that 26 out of 60 bat species in the Caribbean cohabit in urban and peri-urban areas, potentially facilitating the dissemination and transmission of zoonotic viruses. The recent report of flavivirus presence in a synanthropic bat species in a Havana municipality [87] further emphasizes the need for research on TBVs.

Understanding these dynamics, identifying the reservoir bat species, and anticipating potential virus spillover to humans are crucial for developing effective programs in the control and risk assessment of TBVs in public health preparedness.

It is important to note that this review was limited by the scarcity of published primary studies and the insufficient recent information available from many islands in the Caribbean.

## Additional information

No additional information is available for this paper.

## CRediT authorship contribution statement

**Maritza Pupo Antúnez:** Writing – review & editing, Writing – original draft, Supervision, Conceptualization. **José Carlos Marín Montesinos:** Writing – review & editing, Writing – original draft. **Alexandra Corduneanu:** Writing – review & editing, Writing – original draft. **Dasiel Obregón:** Writing – review & editing, Visualization. **Sara Moutailler:** Writing – review & editing, Writing – original draft. **Alejandro Cabezas-Cruz:** Writing – review & editing, Supervision, Conceptualization.

## Declaration of competing interest

The authors declare that they have no known competing financial interests or personal relationships that could have appeared to influence the work reported in this paper.
